# Prognostic value of pan-immune-inflammation in breast cancer patients with lymph node metastasis and neoadjuvant therapy

**DOI:** 10.1097/MD.0000000000045713

**Published:** 2025-11-21

**Authors:** Zengyan Wang, Xiong Wu, Leyi Yang, Yujuan Guo, Xiaoxu Zeng, Xin Yan, Jianhui Chen

**Affiliations:** a Department of Breast Surgery, Zhangzhou Municipal Hospital of Fujian Province and Zhangzhou Affiliated Hospital of Fujian Medical University, Zhangzhou, Fujian Province, People’s Republic of China.

**Keywords:** biomarker, breast cancer with lymph node metastasis, neoadjuvant therapy, pan-immune-inflammation value, prognosis

## Abstract

This study aimed to evaluate the prognostic value of the pan-immune-inflammation value (PIV) in breast cancer patients with lymph node metastasis receiving neoadjuvant therapy. A total of 144 patients treated at our hospital between January 2019 and December 2022 were retrospectively analyzed. PIV was calculated from baseline blood counts obtained before neoadjuvant chemotherapy. Using X-tile software, the optimal cutoff value of 187 was identified, and patients were classified into high (≥187) and low (<187) PIV groups. Progression-free survival (PFS) was assessed using the Kaplan–Meier method and log-rank test, and multivariate Cox regression was performed to determine independent prognostic factors. Predictive accuracy was further evaluated with receiver operating characteristic curves. Patients with high-PIV values had significantly shorter PFS compared with those in the low-PIV group (*P* = .002). Multivariate Cox regression confirmed PIV as an independent prognostic factor. Receiver operating characteristic curve analysis showed that PIV had good predictive performance for 1-, 2-, and 3-year PFS, with AUCs of 0.867, 0.802, and 0.853, respectively. PIV was associated with prognosis in breast cancer patients with lymph node metastasis undergoing neoadjuvant therapy and demonstrated favorable predictive performance in this study. Nevertheless, as this was a single-center retrospective analysis with limited sample size, further validation in larger prospective multicenter studies is necessary before clinical application.

## 1. Introduction

Breast cancer is the most common cancer among women worldwide and remains a leading cause of cancer-related death.^[1]^ Surgery is the cornerstone of treatment for patients with early-stage disease, whereas neoadjuvant chemotherapy (NAC), or preoperative chemotherapy, has become a standard option for locally advanced breast cancer due to its ability to reduce tumor burden, improve surgical outcomes, and prolong survival.^[2]^ However, the response to NAC varies considerably among patients,^[3,4]^ highlighting the urgent need for reliable biomarkers to guide patient selection and optimize therapeutic strategies.

Conventional serum tumor markers, such as CA153, CA125, and CEA, may provide some diagnostic information, but their prognostic performance is limited due to the heterogeneity of breast cancer subtypes.^[5]^ In recent years, increasing attention has been paid to inflammation-associated tumor biology and the tumor microenvironment.^[6–9]^ Chronic inflammation has been shown to promote cancer progression, and peripheral blood immune cells can serve as accessible indicators of systemic immune-inflammation status.^[10–12]^

Among the emerging biomarkers, the pan-immune-inflammation value (PIV), calculated from neutrophils, monocytes, lymphocytes, and platelets, has been reported to reflect systemic inflammation and immune activation. PIV has demonstrated prognostic value in several solid tumors, including breast and colorectal cancers.^[13–19]^ Compared with other biomarkers, PIV offers the advantages of simplicity, low cost, and routine availability in clinical practice. Recent studies suggest that lower PIV values are associated with better therapeutic responses in breast cancer patients undergoing NAC.^[18]^

Although risk prediction models and radiomics-based nomograms for breast cancer outcomes have been reported in the literature, most are focused on radiological or imaging features rather than routine hematological markers. To date, no studies have specifically evaluated the prognostic significance of PIV in breast cancer patients with lymph node metastasis undergoing NAC, despite their higher risk of recurrence and progression due to unique immune-inflammatory profiles.^[20,21]^

Therefore, the aim of this study was to investigate the prognostic value of PIV in breast cancer patients with lymph node metastasis receiving neoadjuvant therapy, and to explore its potential as a simple, efficient, and cost-effective biomarker for prognostic evaluation.

## 2. Materials and methods

### 2.1. Study design

This study is a retrospective single-center study, selecting 144 patients with breast cancer accompanied by lymph node metastasis who underwent neoadjuvant therapy in our hospital from January 2019 to December 2022 as the research subjects. The inclusion criteria were as follows: age ≥18 years old; pathologically confirmed breast cancer with lymph node metastasis; peripheral blood data including neutrophil, monocyte, lymphocyte, and platelet counts collected before neoadjuvant chemotherapy (NAC); complete clinical information of other patients. The exclusion criteria were as follows: age <18 years old; receiving immunomodulatory therapy or neoadjuvant endocrine therapy; history of malignant tumors in other parts of the body; history of hematologic diseases; history of infectious diseases such as HIV; incomplete patient data. Other potential confounding factors such as nutritional status, socioeconomic level, and comorbidities were not systematically assessed and therefore were not included in the exclusion criteria. Ethical approval was obtained by the Clinical Trial Ethics Committee of Zhangzhou Municipal Hospital of Fujian Province (Ethical approval No.: 2023LWB313), and the study was conducted in strict accordance with the ethical principles involving human subjects in medical research outlined in the Helsinki Declaration. Informed consent was obtained from patients who were still alive at the time of data collection and/or analysis for the use of their data for research purposes.

### 2.2. Study objectives

This study aims to retrospectively analyze the correlation between pre-NAC PIV values and prognosis indicators such as progression-free survival (PFS). All patients were followed up until death, loss to follow-up, or data lock (March 31, 2024).

### 2.3. Efficacy indicators

We collected clinical data, including absolute counts of neutrophils, lymphocytes, platelets, and monocytes in peripheral blood, from patients who met the inclusion criteria. All blood samples were obtained within 7 days prior to the initiation of neoadjuvant chemotherapy and before the administration of any systemic therapy. Samples were collected in the morning after overnight fasting and analyzed according to standard hospital laboratory protocols. Only baseline values obtained before the first cycle of neoadjuvant chemotherapy were used for PIV calculation. According to the definition of PIV, PIV = neutrophil count (10^3^/mmc) × platelet count (10^3^/mmc) × monocyte count (10^3^/mmc)/ lymphocyte count (10^3^/mmc).^[19]^ X-tile 3.6.1 software was used to determine the optimal threshold of PIV values corresponding to the maximum difference in the Kaplan–Meier (KM) curve. According to the optimal threshold of PIV, patients were divided into high-PIV group and low-PIV group.

### 2.4. Statistical methods

Descriptive statistics were used to analyze patient characteristics. Univariate and multivariate Cox regression analyses were used to analyze factors affecting PFS. The KM method was used to draw PFS survival curves, and the log-rank test was used to compare the survival distributions of different patient groups. Fischer exact test was used for categorical variables, and an independent t-test was used for continuous variables. Variables with a *P*-value <.10 in univariate analysis were entered into the multivariate Cox regression model. In addition, clinically relevant factors (such as age, tumor stage, ER, progesterone receptor, and HER2 status) were also considered for inclusion regardless of their univariate significance. All analyses were 2-sided statistical tests, and *P* <.05 was considered statistically significant. Other statistical analyses were performed using SPSS 21.0 statistical software (Chicago).

The required sample size was estimated using PASS software based on survival analysis. Assuming a hazard ratio of 1.8 between the high and low-PIV groups, a 2-sided alpha level of 0.05, and a power of 80%, the minimum sample size was calculated to be 120 patients. Considering a potential dropout or loss to follow-up rate of approximately 15%, the final target sample size was set at 138 patients. Eventually, 144 eligible patients were enrolled, meeting the required statistical power.

## 3. Results

### 3.1. Patient baseline characteristics

The basic characteristics of the patients are summarized in Table 1. A total of 144 cases were included in the analysis, with a median age of 52 years, spanning a range from 27 to 78 years. This demographic indicates a diverse patient population, reflecting various age-related factors that may influence treatment outcomes and prognosis. Additional baseline characteristics such as tumor grade, lymph node involvement, and previous treatments are detailed in the table, providing a comprehensive overview of the patient cohort.

**Table 1:** Clinical and pathological characteristics of patients.

Patient characteristics	N (144)	%
Age		
Median (min–max)	52 (27–78)	100
Clinical stage		
T1–T2	88	61.1
T3–T4	56	38.9
Histopathology		
Invasive ductal carcinoma	141	97.9
Other types	3	2.1
Estrogen receptor		
Negative	60	41.7
Positive	84	58.3
HER2 status		
Negative	76	52.8
Positive	68	47.2
Ki67 index (%)		
Median (Range)	30 (1–90)	100
Chemotherapy regimen		
Anthracycline plus Taxane	112	77.8
Anthracycline-based regimen	1	0.7
Taxane-based regimen	31	21.5
Lost to follow-up	5	3.5
Missing values	0	0

HER2 = human epidermal growth factor receptor 2.

### 3.2. Optimal threshold for PIV

The optimal threshold for the pan-immune-inflammation value (PIV) was determined to be 187, utilizing X-tile 3.6.1 software. This software helps identify the maximum difference in the KM survival curves associated with varying PIV values. Based on this analysis, participants were stratified into 2 groups: a high-PIV group (≥187) and a low-PIV group (<187). This threshold is critical for clinical decision-making, as it enables stratification of patients based on their inflammatory profiles, which can influence treatment approaches and prognostic assessments.

### 3.3. Univariate and multivariate cox regression analysis

To further investigate the impact of PIV on PFS, we conducted univariate and multivariate logistic regression analyses. The univariate analysis included various clinical indicators related to breast cancer and PIV. The results revealed that factors such as T stage, histological type, estrogen receptor (ER), progesterone receptor, HER2 status, Ki67 proliferation index, and PIV were all potential predictors associated with PFS (Table 2).

**Table 2:** Univariate and multivariate Cox regression analysis.

Patient characteristics	Univariate analysis (HR, 95% CI)	*P*-value	Multivariate analysis (HR, 95% CI)	*P*-value
Age (years) (≥53 vs <53)	1.163 (0.976–1.234)	.684	–	–
Clinical T stage (T1T2 vs T3T4)	1.893 (1.475–3.046)	<.001*	1.571 (1.065–2.643)	.026*
Histological type (invasive ductal carcinoma vs others)	2.782 (1.269–4.215)	<.001*	2.462 (1.138–4.062)	<.001*
ER (negative vs positive)	0.621 (0.402–0.841)	.006*	0.921 (0.612–1.632)	.698
PR (negative vs positive)	0.573 (0.422–0.861)	.007*	0.881 (0.368–1.264)	.065
HER2 (negative vs positive)	1.545 (1.122–2.126)	.002*	1.216 (0.863–1.954)	.129
Ki67 (≥38 vs <38)	2.438 (1.426–3.263)	<.001*	1.926 (1.079–2.651)	.042
PIV value (≥187 vs<187)	1.821 (1.120–2.638)	.012*	1.635 (1.036–2.865)	.039*

CI = confidence interval, ER = estrogen receptor, HER2 = human epidermal growth factor receptor 2, HR = hazard ratio, PIV = pan-immune-inflammation value, PR = progesterone receptor.

* Indicates statistical significance.

Subsequently, we performed multivariate Cox regression analysis to isolate the independent prognostic factors affecting PFS. This analysis demonstrated that T stage, histological type, Ki67, and PIV emerged as significant independent prognostic factors. The identification of these variables as significant highlights their importance in clinical settings, suggesting that they can guide treatment strategies and patient management plans.

### 3.4. Impact of PIV Value on PFS

**Figure 1.**
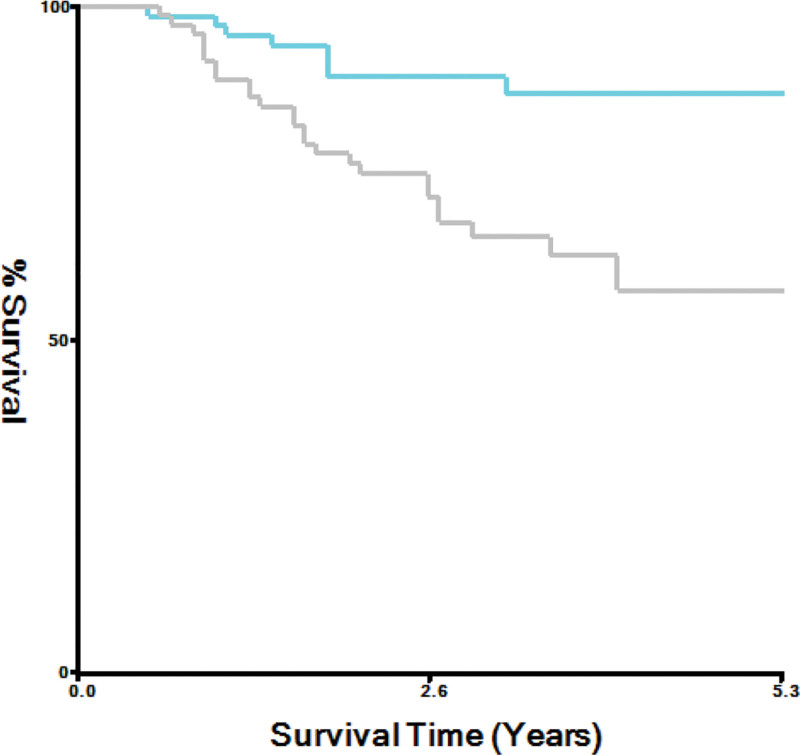
Kaplan–Meier plots of PFS endpoints in low-PIV and high-PIV groups. The bright green curve indicates low-PIV group and the gray one represents high-PIV group. Please note that color should be used for this figure in print. PFS = progress-free-survival, PIV = pan-immune-inflammation value.

The median follow-up time for the study cohort was 30.2 months, during which a total of 34 cases of disease progression were recorded. To illustrate the survival differences between the 2 groups, KM curves were generated, and log-rank tests were conducted. These analyses indicated a statistically significant difference in PFS between patients with low-PIV values and those with high-PIV values, with *P* = .002 (Fig. 1). Specifically, patients in the low-PIV group demonstrated significantly longer PFS times compared to their counterparts in the high-PIV group.

### 3.5. Prognostic value of PIV in predicting 1-year, 2-year, and 3-year PFS

To further assess the prognostic utility of PIV, we plotted receiver operating characteristic curves to evaluate its predictive accuracy for 1-year, 2-year, and 3-year PFS in breast cancer patients with lymph node metastasis. The area under the curve (AUC) was calculated to quantify the discriminatory ability of PIV at each time point.

**Figure 2.**
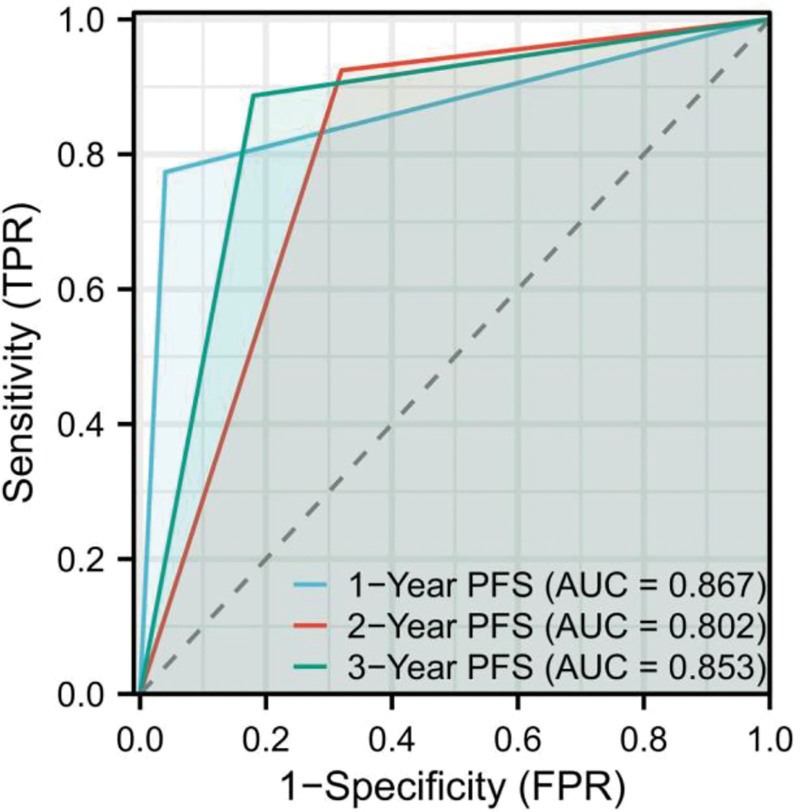
ROC curves illustrating the predictive value of PIV for 1-yr, 2-yr, and 3-yr PFS in breast cancer patients with lymph node metastasis. The AUC for 1-yr PFS, 2-yr PFS, and 3-yr PFS were 0.867, 0.802, and 0.853, respectively, indicating that PIV is a strong prognostic biomarker across different time points. AUC = area under the curve, PFS = progress-free-survival, PIV = pan-immune-inflammation value.

The results showed that the AUC for 1-year PFS was 0.867, indicating a high level of accuracy in predicting short-term outcomes. Similarly, the AUC values for 2-year and 3-year PFS were 0.802 and 0.853, respectively (Fig. 2).

## 4. Discussion

In recent years, there has been a growing focus on the prognostic factors and related biomarkers of breast cancer, driven by the urgent need to enhance prevention and treatment strategies as well as clinical outcomes. The identification of a simple, feasible, cost-effective, and reliable biomarker is crucial for clinical practice and the prognostic assessment of patients. Peripheral blood analysis, specifically the proportions of different cell types, can provide valuable insights into systemic inflammatory and immune levels, which can influence prognosis to a certain extent.^[12,22–24]^ Numerous studies have demonstrated a strong correlation between systemic inflammatory and immune levels and the occurrence, progression, and treatment strategies of tumors.^[25]^ This relationship not only highlights the complexity of cancer biology but also directs research toward identifying prognostic biomarkers derived from blood samples.

Research has increasingly indicated that immune cells, such as neutrophils, can reflect systemic inflammation or immune suppression status and are significantly associated with the prognosis of tumor immunotherapy.^[26]^ This is particularly relevant as the immune landscape of cancer patients plays a critical role in determining treatment responses and outcomes. Understanding the immune profile can inform personalized treatment approaches, potentially improving efficacy and reducing adverse effects.

While previous studies have shown that breast cancer patients with lymph node metastasis often exhibit elevated levels of inflammation and a unique immune tumor microenvironment,^[21,22,27–29]^ it is noteworthy that lower Pan-Immune-Inflammation Value (PIV) has been associated with better prognostic outcomes for neoadjuvant therapy in breast cancer. Given the distinctive tumor immune microenvironment in patients with lymph node metastasis, it is hypothesized that PIV may serve as an important prognostic biomarker for neoadjuvant therapy in this subset of patients.^[18]^ Our study is the first to reveal the impact of pre-neoadjuvant chemotherapy (NAC) PIV values on the prognosis of breast cancer patients with lymph node metastasis. The findings indicate that patients with low pre-NAC PIV values experience a longer PFS following neoadjuvant therapy, suggesting that PIV values can be utilized for effective prognostic assessment. The final enrollment of 144 patients exceeded the minimum sample size calculated.

The implications of our research extend beyond mere statistical associations; they may reflect, on a microscopic level, the presence of similar peripheral blood immune cell types and combinations in populations sharing the same or similar high-risk profiles. This can manifest macroscopically as an overall immune suppression status and a correlated poor prognosis in the body. As we delve deeper into the dynamics of the tumor microenvironment, an increasing number of studies emphasize its significance in cancer biology. Many clinical trials are being conducted to enhance breast cancer efficacy by modifying the tumor microenvironment, particularly targeting immune-inflammatory cells and related factors.^[30,31]^ This focus on the tumor microenvironment not only deepens our understanding of cancer progression but also opens new avenues for therapeutic interventions.

Moreover, our study presents PIV as a practical biomarker that could bridge the gap between clinical practice and research. Previous investigations have established that PIV values serve as independent prognostic factors for various cancers, including colorectal cancer, breast cancer, non-small cell lung cancer, and gastric cancer. These values are recognized as simple and feasible clinical indicators for predicting outcomes such as PFS and overall survival (OS) in cancer patients, often demonstrating higher prognostic value than other established immune-inflammatory biomarkers like neutrophil-to-lymphocyte ratio.^[32–34]^ The ability to use PIV as a routine clinical indicator not only simplifies the process for healthcare providers but also enhances patient management through timely interventions.

In summary, PIV demonstrates significant clinical potential, warranting further exploration and validation. However, it is important to note that our study is based on retrospective data from a single center, with a relatively small sample size and a short follow-up period. This single-center design ensures uniformity in patient treatment and follow-up, leading to more reproducible laboratory data collection. Nevertheless, larger sample sizes and prospective, randomized, double-blind, multicenter clinical trials are necessary to validate the clinical value of PIV fully. In addition, due to the limited number of cases, we were unable to perform subgroup analyses according to different breast cancer subtypes (e.g., TNBC, HER2+), which may influence the prognostic performance of PIV. Moreover, although our findings support the prognostic utility of PIV, the underlying biological mechanisms linking systemic immune-inflammation status and treatment outcomes were not fully elucidated in this study. Furthermore, other potential confounding factors, such as nutritional status, socioeconomic level, and comorbidities, were not systematically assessed, which may have influenced the results. Finally, only internal bootstrap validation was performed in this study, and no external dataset was available for validation. Thus, future studies should include external validation cohorts or supplementary sensitivity analyses to confirm the robustness and generalizability of our findings. Future prospective multicenter studies integrating clinical, molecular, and immunological data are warranted to validate our findings, assess the applicability of PIV across different breast cancer subtypes, and provide deeper mechanistic insights into the link between systemic immune-inflammation and treatment outcomes.

Furthermore, future research should aim to assess the applicability of PIV in different molecular subtypes of breast cancer, such as TNBC and HER2-positive disease, since tumor biology may significantly influence PIV levels and their prognostic significance. In addition, investigating the potential synergistic effects of PIV in combination with other hematological or molecular biomarkers may provide a more comprehensive evaluation of its clinical utility. By addressing these aspects, future studies can uncover deeper insights into the biological mechanisms linking systemic immune-inflammation and treatment outcomes, ultimately contributing to more effective therapeutic strategies and improved prognostic assessment for breast cancer patients.

## Acknowledgments

Thanks to the nurses in the department for their help with the project.

## Author contributions

**Conceptualization:** Zengyan Wang, Xiong Wu, Leyi Yang, Yujuan Guo, Xiaoxu Zeng, Xin Yan, Jianhui Chen.

**Data curation:** Zengyan Wang, Xiong Wu.

**Formal analysis:** Zengyan Wang, Leyi Yang.

**Investigation:** Zengyan Wang, Yujuan Guo, Xiaoxu Zeng.

**Methodology:** Zengyan Wang, Xin Yan, Jianhui Chen.

**Supervision:** Zengyan Wang, Xiong Wu, Leyi Yang, Yujuan Guo, Xiaoxu Zeng, Xin Yan, Jianhui Chen.

**Validation:** Zengyan Wang, Xiong Wu, Leyi Yang, Yujuan Guo, Xiaoxu Zeng, Xin Yan, Jianhui Chen.

**Visualization:** Zengyan Wang, Xiong Wu, Leyi Yang, Yujuan Guo, Xiaoxu Zeng, Xin Yan, Jianhui Chen.

**Writing – original draft:** Zengyan Wang, Xiong Wu.

**Writing – review & editing:** Zengyan Wang, Xiong Wu.
